# Supplementary material for: Analysis of PPARγ Signaling Activity in Psoriasis
Source: Int J Mol Sci. 2021 Aug 10;22(16):8603. doi: 10.3390/ijms22168603 (PMC8395241; doi:10.3390/ijms22168603)
Supplement: Supplementary file 1 [file ijms-22-08603-s001.zip › Supplemental materials_Analysis of PPARg signaling activity in psoriasis/Pathway models/Models images and html files/PPARG expressed in cells assotiated with psoriasis/225624.html]

antigen-presenting cell


# CellType antigen-presenting cell

|  |  |
| --- | --- |
| URN | urn:agi-ncimcelltype:C0003315 |
| Total Entities | 40 |
| Connectivity | 2262 |
| Name | antigen-presenting cell |

---

|  |  |
| --- | --- |
| ChildConcepts | dendritic cell |
|  | phagocyte |
|  | antigen-presenting cell function |
|  | antigen-presenting cell infiltration |
|  | antigen-presenting cell differentiation |
|  | antigen-presenting cell formation |
|  | antigen-presenting cell development |
|  | antigen-presenting cell migration |
|  | antigen-presenting cell adhesion |
|  | antigen-presenting cell population |
|  | antigen-presenting cell interaction |
|  | antigen-presenting cell phenotype |
|  | antigen-presenting cell contact |
|  | antigen-presenting cell count |
|  | antigen-presenting cell proliferative response |
|  | antigen-presenting cell damage |
|  | antigen-presenting cell polarity |
|  | antigen-presenting cell homing |
|  | antigen-presenting cell priming |
|  | antigen-presenting cell survival |
|  | antigen-presenting cell chemotaxis |
|  | antigen-presenting cell apoptosis |
|  | antigen-presenting cell proliferation |
|  | antigen-presenting cell growth |
|  | antigen-presenting cell compartmentalization |
|  | antigen-presenting cell sequestration |
|  | antigen-presenting cell death |
|  | antigen-presenting cell destruction |
|  | antigen-presenting cell localization |
|  | antigen-presenting cell recognition |
|  | antigen-presenting cell phagocytosis |
|  | antigen-presenting cell cytotoxicity |
|  | antigen-presenting cell homeostasis |
|  | antigen-presenting cell extravasation |
|  | antigen-presenting cell selection |
|  | antigen-presenting cell transendothelial migration |
|  | antigen-presenting cell repertoire |
|  | antigen-presenting cell clonal anergy |
|  | antigen-presenting cell motion |
|  | antigen-presenting cell mediated cytotoxicity |

---

|  |  |
| --- | --- |
| Pathway | IL23A and IL17A Provoke Cancer-Associated Inflammation |
|  | IL12 Antitumor Effect is Blocked in Cancer |
|  | Adenosine/cAMP Promote Immunosuppression by Treg Cells |
|  | PPARG expressed in cells assotiated with psoriasis |
|  | Neighbors of antigen-presenting cell |

---

|  |  |
| --- | --- |
| MedScan ID | 10004000 |

---

|  |  |
| --- | --- |
| Alias | immunologic accessory cells |
|  | immunologic accessory cell |
|  | antigen-presenting cell |
|  | antigen-presenting cells |

---

|  |  |
| --- | --- |
| NCIm ID | C0003315 |

---
